# Supplementary material for: The causal role of circulating inflammatory markers in osteoporosis: a bidirectional Mendelian randomized study
Source: Front Immunol. 2024 Jul 18;15:1412298. doi: 10.3389/fimmu.2024.1412298 (PMC11291241; doi:10.3389/fimmu.2024.1412298)
Supplement: Supplementary file 1 [file DataSheet_1.zip › supplementary material/OSTEOPOROSIS-CIM/GCST90274766.tsv.gz_leave_one_out.pdf]

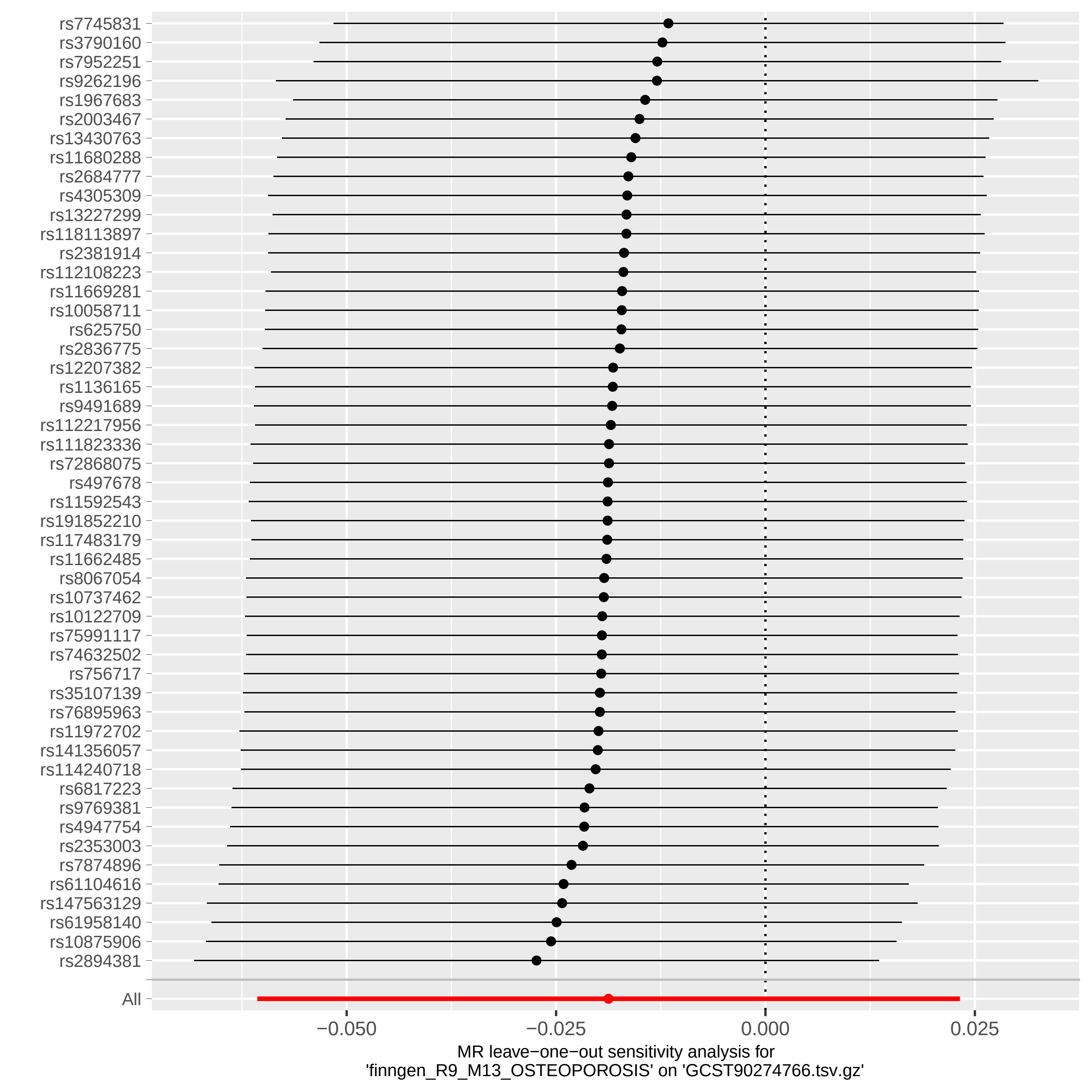
rs7745831
rs3790160
rs7952251
rs9262196
rs1967683
rs2003467
rs13430763
rs11680288
rs2684777
rs4305309
rs13227299
rs118113897
rs2381914
rs112108223
rs11669281
rs10058711
rs625750
rs2836775
rs12207382
rs1136165
rs9491689
rs112217956
rs111823336
rs72868075
rs497678
rs11592543
rs191852210
rs117483179
rs11662485
rs8067054
rs10737462
rs10122709
rs75991117
rs74632502
rs756717
rs35107139
rs76895963
rs11972702
rs141356057
rs114240718
rs6817223
rs9769381
rs4947754
rs2353003
rs7874896
rs61104616
rs147563129
rs61958140
rs10875906
rs2894381
All
−0.050
−0.025
0.000
0.025
MR leave−one−out sensitivity analysis for
'finngen_R9_M13_OSTEOPOROSIS' on 'GCST90274766.tsv.gz'
